# Characterization of prokaryotic communities in Puerto Rican caves using 16S rDNA amplicon sequencing

**DOI:** 10.1128/mra.00354-25

**Published:** 2025-06-10

**Authors:** Natalia Pérez-Santos, Sebastian Javier Borrero-Villabol, Rene Nieves-Morales, Jessica Alejandra Paez-Díaz, Edwin Omar Rivera-Lopez, Josué Rodríguez-Ramos, Angel M. Nieves-Rivera, Carlos Ríos-Velazquez

**Affiliations:** 1 Microbial Biotechnology and Bioprospecting Laboratory, Biology Department, University of Puerto Rico, Mayagüez, USA; 2 Department of Food Science, The Pennsylvania State University 8082, University Park, Pennsylvania, USA; 3 Pacific Northwest National Laboratory, Biological Sciences Division 6865, Richland, Washington, USA; 4 Grupo Espeleológico del Oeste, Inc. (GEO), Mayagüez, Puerto Rico; University of Maryland School of Medicine, Baltimore, Maryland, USA

**Keywords:** 16S rDNA, amplicons, Puerto Rico, caves, sequencing, prokaryotes

## Abstract

The cave ecosystems host microbial communities adapted to extreme environments. This study utilized 16S rDNA to investigate the prokaryotic diversity across seven caves in Puerto Rico’s northern limestone karst belt. Microbial profiling revealed distinct subterranean communities, enhancing our understanding of cave microbiology and potential applications in environmental conservation and microbial research.

## ANNOUNCEMENT

Caves are characterized by unique conditions such as low light, moisture, restricted airflow, and variable gas concentrations, which sustain diverse microbial communities (1). These microorganisms play a critical role in biosphere processes, such as nitrogen fixation and organic carbon mineralization (2). Additionally, microbes contribute to biochemical cycling, like iron oxidation, manganese, and nitrates (3). Due to limited anthropogenic impact, caves support the natural development of specialized microbes in which recent advances in molecular biology and geochemistry have made possible the study of these microbial communities through culture-independent techniques (4), revealing microorganisms that may harbor novel biological compounds relevant to biomedical applications, including antibiotics, antifungal, and antiviral compounds. Caves in Puerto Rico’s northern limestone karst belt area are typically acidic and humid ecosystems (5, 6). These microbial profiles enhance taxonomical knowledge of local cave environments, improving our understanding of the microbial diversity and ecological dynamics within this relatively unexplored environment. Seven caves in the northern limestone karst belt area of Puerto Rico were sampled. The dates and locations of the samples collected are presented in (Table 1). Using a sterile Whirl–Pak bag, 30 g of superficial bat guano-enriched soil was obtained from the dark zones of each cave (7, 8).

**TABLE 1:** Cave localization and sampling dates

Sampled caves	Municipality	Coordinates	Collection date
Cueva Alayón	Las Marías	18°13'38.8"N, 66°59'49.4"W	Sept/14/2024
Cueva Ensueño	Hatillo	18°19'29"N, 66°49'28"W	Dec/09/2023
Cueva Clara	Hatillo	18°20'38"N, 66°49'19"W	Feb/25/2024
Cueva de los Panes	Utuado	18°17'50"N, 66°44'24"W	Sept/28/2024
Cueva León	Arecibo	18°22'20"N, 66°41'22"W	Jan/02/2024
Cueva Catedral I–II	Florida	18°20'35.3"N, 66°31'46.7"W	Jun/29/2024
Cueva Oscura	Aguas Buenas	18°13'38"N, 66°06'29.9"W	Apr/07/2024

Direct DNA extractions were performed utilizing 10 g of guano-enriched soil. This process included chemical (Sodium Dodecyl Sulfate [SDS] and Guanidine Isothiocyanate [GITC]), mechanical (freeze-thaw), and enzymatic (lysozymes) approaches, following the protocol described by Rivera-Lopez et al. (9) (10). DNA purification was performed by gel electrophoresis, followed by extraction of the desired fragment (1-25kb) following the GELase protocol (CopyControl Fosmid Library Production Kit with pCC1FOS Vector, Lucigen). The resulting DNA was sent to the Molecular Research Laboratory, MR. DNA (https://www.mrdnalab.com/), where sequencing was performed using the Illumina MiSeq system, amplifying for the V3–V4 regions using the 341F (CCTACGGGNGGCWGCAG) and 805R (GACTACHVGGGTATCTAATCC) primers (9). The library concentration was reduced to 4.0 nM, samples were fragmented, and adapter sequences were added. Sequencing was performed with 2 × 300 paired end reads for 30 cycles (11). The data were analyzed using QIIME-2 (amplicon-2024.5) (12) with default parameters, unless otherwise noted. DADA2 (version 1.30.0) (13) was used to denoise single-end reads, with the flag “p-trunc-len 150” for trimming. Cutadapt was used to remove primers (14). This resulted in 972,680 quality reads for microbial taxa analysis. Afterward, a total of 874,565 non-chimeric sequences were assessed, averaging 84.24% of the resulting denoised sequences. The data were clustered into 5,718 ASVs at a 100% similarity threshold.

**Fig 1:**
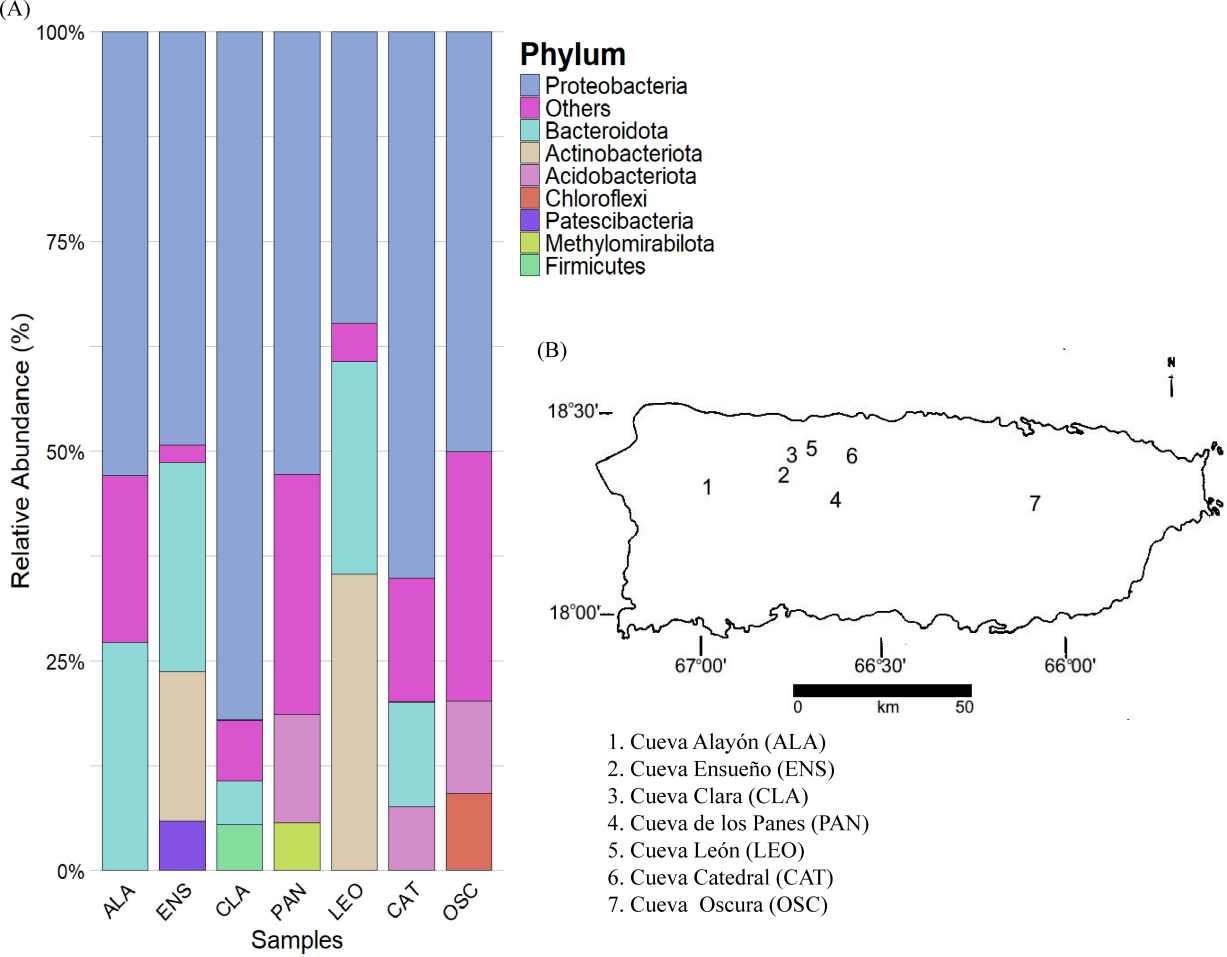
Relative abundance of prokaryotic communities at the phylum level across seven Puerto Rican caves. (A) Taxa bar plots displaying the relative abundance of microbial phyla across the samples; phyla contributing less than 5% to the total relative abundance are aggregated into a single category labeled “others.” (B) Map of Puerto Rico indicating the locations of the sampled caves. The map was generated by the author, Angel Nieves.

A total of 49 phyla were detected, and composition across the samples (Fig. 1) was distributed as follows: ALA was dominated by Proteobacteria (52.59%) and Bacteroidota (27.096%). ENS was mostly composed of Proteobacteria (37.751%), Actinobacteria (13.649%), and Bacteroidota (19.152%). CLA by Proteobacteria (78.095%), Actinobacteria (4.708%), and Bacteroidota (12.303%). PAN was dominated by Proteobacteria (50.848%) and Actinobacteria (12.441%). LEO of Proteobacteria (32.841%), Actinobacteria (33.381%), and Bacteroidota (23.873%). CAT was mainly Proteobacteria (63.978%) and Bacteroidota (12.303%). Finally, OSC featured Proteobacteria (48.225%) and Actinobacteria (10.603%).

## Data Availability

The raw data from this project has been deposited in the National Center for Biotechnology Information (NCBI) under the BioProject accession number PRJNA1232682. The samples were distributed as follows: Cueva Alayón (ALA) (barcode: ATCGATGC; SRA accession number: SRS23684827), Cueva Ensueño (ENS) (barcode: ATCGCCTT; SRA accession number: SRS23684828), Cueva Clara (CLA) (barcode: ATCGATGC; SRA accession number: SRS23684829), Cueva de los Panes (PAN) (barcode: ATCGCGAT; SRA accession number: SRS23684830, Cueva León (LEO) (barcode: ATCGCCAA; SRA accession number: SRS23684831), Cuevas Catedral I–II (CAT) (barcode: ATCGCCAA; SRA accession number: SRS23684832), Cueva Oscura (OSC) (barcode: ATCGCCTT: SRA accession number: SRS23684833).
